# Potentiation and Electrical Stimulus Frequency During Self-Paced Exercise and Recovery

**DOI:** 10.2478/hukin-2014-0064

**Published:** 2014-10-10

**Authors:** Christian Froyd, Fernando G. Beltrami, Jørgen Jensen, Guillaume Y. Millet, Timothy David Noakes

**Affiliations:** 1 UCT/MRC Research Unit for Exercise Science and Sports Medicine, Department of Human Biology, University of Cape Town, South Africa.; 2 Faculty of Teacher Education and Sport, Sogn og Fjordane University College, Norway.; 3 Department of Physical Performance, Norwegian School of Sport Sciences, Norway.; 4 The University of Calgary, Faculty of Kinesiology, Calgary, Canada.

**Keywords:** Electrical stimulation, twitch, peripheral fatigue, potentiation, time trial

## Abstract

The aim of this study was to investigate the effect of potentiation on stimulation-induced muscle function during and after an intense bout of self-paced dynamic exercise. Ten active subjects performed a time trial involving repetitive concentric extension-flexion of the right knee using a Biodex dynamometer. Electrical stimulation before and after a 5 s maximal isometric voluntary contraction was performed before the start of the time trial and immediately (< 5 s) after each 20% of the time trial as well as 1, 2, 4 and 8 min after time trial termination. Potentiation was observed before the time trial and as early as 1–2 min after the time trial, but no potentiation was detected during or immediately after the time trial for neither single or paired stimuli. At termination of the time trial, “potentiated” peak torque was significantly more reduced than “unpotentiated” peak torque for single stimulus (−65 ± 10% and −42 ± 18%, respectively) and paired stimuli at 100 Hz (−51 ± 10% and −33 ± 15%, respectively). Faster recovery for “potentiated” compared to “unpotentiated” peak torque indicate that potentiate peak torque measurements or delay the post-exercise measurements more than a few seconds, will underestimate peripheral fatigue. In conclusion, the potentiation after maximal contraction disappears during intense exercise. Whether the muscle is already potentiated during intense contraction or fatiguing mechanisms inhibits potentiation remains to be clarified.

## Introduction

Post activation potentiation, subsequently termed potentiation, is defined as the increased response to electrical stimulation (ES) after an initial voluntary contraction (Vandervoort et al., 1983; McComas et al., 1983; Sale, 2002; Moore and Stull, 1984). Potentiation is explained by increased Ca^2+^ sensitivity mainly due to increased myosin regulatory light chain (RLC) phosphorylation (Macintosh et al., 2012), which increases muscle contractility. The magnitude of potentiation is striking because it can instantly increase evoked knee extensor force output by 40–60% (Green and Jones, 1989; Paasuke et al., 2007; Requena et al., 2008; Froyd et al., 2013a). This effect is important in studies quantifying peripheral fatigue (Place et al., 2010) in terms of changes in neuromuscular function (NMF) in response to ES during or after exercise. Accordingly, we define peripheral fatigue as a reduction in the peak evoked torque (PT) in response to ES on relaxed muscles.

When the muscles’ ability to produce force is re-measured in the post-exercise period, the change from the pre-exercise value will be the result of two opposing forces – potentiation which will increase -and repeated contractions which will induce fatigue and decrease PT in response to ES. It is not known to what extent either contributes to the change in PT measured after the exercise bout (Rassier and Macintosh, 2000). Several studies investigating the development of peripheral fatigue during prolonged (Millet et al., 2002; Gauche et al., 2006) or short duration (Skof and Strojnik, 2006; Gondin et al., 2006; Skurvydas et al., 2008) exercise however have performed the pre-exercise measurement in muscles that were not exposed to potentiation. Other studies have attempted to differentiate between potentiation and peripheral fatigue during exercise, but without measuring the maximal possible potentiation before exercise began (Fowles and Green, 2003; Morana and Perrey, 2009).

Previous studies have shown that potentiation disappears rapidly after a single maximal voluntary contraction (MVC) of the knee extensors (Green and Jones, 1989; Hamada et al., 2000; Froyd et al., 2013a). However, as far as we know, no study has yet reported the effects of potentiation kinetics during and after (i.e. during the recovery process) a bout of intense dynamic exercise. Also, the contractile response occurring as a result of different types of ES during exercise is unknown. Thus, this study was designed to compare different types of ES on NMF in muscles before and immediately after a 5 s isometric MVC before, during and after a high intensity time trial (TT) involving one-legged dynamic exercise in order to quantify the opposing effect of pre-exercise potentiation and the peripheral fatigue that develops during exercise. We hypothesized that the measurement of the extent of both potentiation and peripheral fatigue would differ with the type of ES used. In addition, we evaluated skeletal muscle contractile characteristics in order to explore the possible mechanical explanations for these phenomena.

## Material and Methods

### Participants

10 physically active (training > 4 times a week) subjects (2 women and 8 men) volunteered to participate in the study. Their average (± SD) age, body mass and height were 23.1 ± 6.0 years, 74.7 ± 9.0 kg, and 180.2 ± 9.0 cm, respectively. The subjects gave their written informed consent to participate in the study, after which they completed a health screening questionnaire. Subjects were given a full explanation of the details and rationale of the study and were informed that they were free to withdraw from the study at any time. The possibility that ES might cause discomfort was fully explained as was the nature of the risks involved. The study was approved by the Ethics Committee of the University of Cape Town, and the experiments were performed according to the latest (2008) revision of the Declaration of Helsinki.

### Experimental design

The applied methods are described in detail in Froyd et al. (2013b). A short description of the methods is as follows: subjects made two preliminary visits to the laboratory during the 3 weeks immediately before the experiments commenced. During both visits, the subjects were familiarized by (I) performing the TT with knee extension-flexion of the right leg and (II) measuring NMF using a Biodex System 3 isokinetic dynamometer (Biodex Medical System, Shirley, NY). Pilot and familiarization testing found that the expected TT time was 4–8 min. NMF was assessed before, during and after the TT.

### Protocol

**Figure 1:**
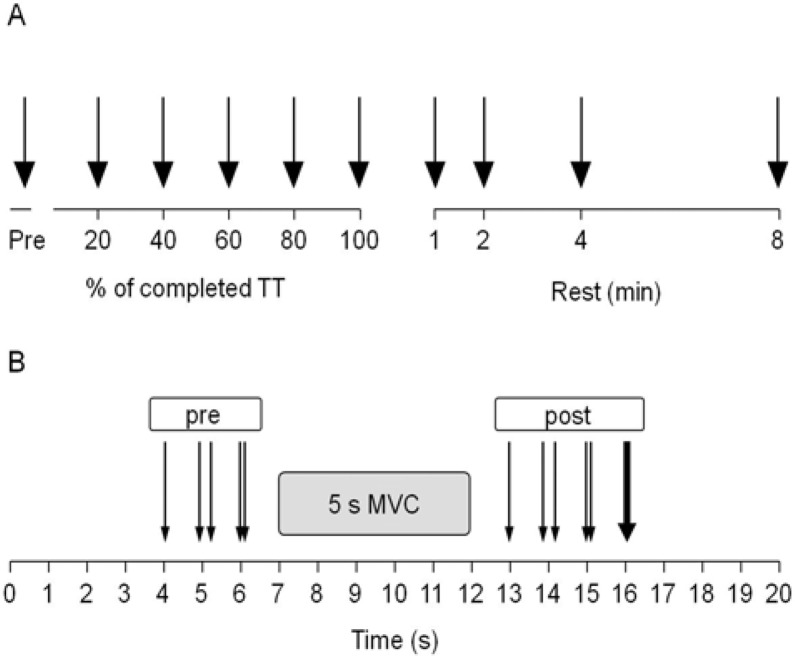
Overview of the stimulation timing and methods during the time trial (TT) (A) and the neuromuscular function (NMF) evaluations (B). A; during the TT, black arrows indicate electrical stimulation (ES) and maximal voluntary contraction (MVC) measurements. B; one thin arrow indicates single stimulus (SS); paired thin arrows indicate paired stimuli at 10 Hz (PS10) and paired stimuli at 100 Hz (PS100). A single solid arrow indicates tetanic stimulation. ES was performed before the MVC and after the MVC. ES responses pre-MVC are referred as “pre” (e.g. PT_pre_ for peak evoked torque) in the manuscript to distinguish from post-MVC referred as “post” (e.g. PT_post_). *The figure is modified from Figure 1* in Froyd et al. *(2013b).*

Subjects performed the TT on the isokinetic dynamometer by performing repetitive concentric extension-flexion movements of the right leg as previously described (Froyd et al., 2013b). Termination (100%) of the TT occurred when 30,000 J of work had been completed on the dynamometer (Figure 1A). NMF evaluation (Figure 1B) was performed before the start of the TT and immediately (mean for all subjects 2.8 ± 0.7 s) after each 20% (6000 J) of the TT had been completed, as well as 1, 2, 4 and 8 min after termination of the TT.

During rest after the TT, subjects continued to sit in the dynamometer and were inactive except when performing the NMF evaluations. Pre-TT NMF was assessed twice separated by 1 min after an isometric warm up. Five maximal extension-flexion concentric isokinetic contractions were performed 3–4 min before the start of the TT.

### Electrical stimulation

After detection of the femoral nerve with a ball probe cathode, ES was applied percutaneously via a 10 mm diameter self-adhesive cathode electrode (Kendall Meditrace, USA) pressed manually onto the skin over the femoral nerve. The anode, a 130×80 mm self-adhesive electrode (Cefar-Compex Scandinavia AB, Sweden) was applied to the gluteal fold.

A constant current stimulator (DS7AH, Digitimer, Hertfordshire, UK) delivered a square-wave stimulus of 200 μs duration at a maximum of 400 V. The optimal stimulation intensity for a single stimulus was determined by increasing the current gradually from 10 mA until a plateau in torque (50–115 mA) was reached. The current was then increased by a further 30% (70–150 mA) to ensure supramaximal stimulation. The intensity was kept constant for the same subject for all types of ES. The subjects were instructed to relax fully when the ES was applied.

### Evaluation of neuromuscular function in response to electrical stimulation

As shown in Figure 1B, NMF evaluation consisted of the following sequence of stimuli before the MVC: single stimulus (SS_pre_); paired stimuli at 10 Hz (PS10_pre_); paired stimuli at 100 Hz (PS100_pre_); after a 5 s isometric MVC, SS_post_, PS10_post_, PS100_post_ and a tetanic stimulation at 100 Hz for 350–600 ms were evoked. During MVC, the subjects were instructed to reach maximum torque in 1 s and then to maintain this level for 4 s whilst they received strong verbal encouragement. ES responses pre-MVC are indicated with “pre” (i.e. PT_pre_) in the manuscript, to distinguish from post-MVC measurements indicated with “post” (i.e. PT_post_). The interval between the stimulation techniques and between stimuli and MVCs was 1 s. PowerLab (ADInstruments Pty Ltd, Bella Vista NSW, Australia) was used to trigger the ES. The hip angle was positioned at 110 deg during all experiments, and the knee angle was positioned at 90 deg when isometric NMF was assessed.

### Time trial

The goal of the TT was to complete 30,000 J of work in the fastest time possible. The subjects performed repetitive isokinetic concentric knee extension-flexions at 300 deg·s^−1^. In addition to knee extension, knee flexion was also used to mimic an activity such as cycling. The range of motion was from knee flexion at approximately 120 deg to full knee extension (anatomical zero) at 0 deg. As a result of familiarization testing, the subjects knew the approximate duration of the TT and were therefore able to pace themselves appropriately. After 18, 38, 58, 78 and 98% of the TT, the subjects were asked to report their rating of perceived exertion (RPEs; Borg, 1974). The TT was briefly stopped for NMF evaluation after 20, 40, 60 and 80% of the TT. These measurements were performed again immediately (< 5 s), and 1, 2, 4, and 8 min after completion of the TT (Figure 1A).

### Torque measurements

Right leg torque was measured in the isokinetic dynamometer during both the TT and the NMF evaluation. During the TT, the dynamic maximum and minimum torque for every cycle of both extension and flexion was measured. Torque response from tetanic stimulation and TT concentric torque can be found in Froyd et al. (2013b)*.*

### Experimental variables and data analysis

In addition to PT, the SS torque response was analysed to determine contraction time (CT), which is the time from start of the contraction to PT, the rate of torque development (RTD) which is PT/CT, half relaxation time (½RT) which is the time from PT to 50% decline in PT, and the rate of relaxation (RR) calculated as PT/½RT. The PS10/PS100 torque ratio was calculated as an index of low/high-frequency fatigue (Verges et al., 2009). The comparative difference in PT_pre_ and PT_post_ (Figure 3) was calculated by dividing the absolute PT value for post-MVC by the pre-MVC value.

### Statistical analyses

The data were analysed with Statistica 10.0 (Stat Soft. Inc., Tulsa, OK). Descriptive statistics are presented as means ± SD unless otherwise stated. Repeated-measures ANOVA was used to detect differences over time. A Tukey *post hoc* test was applied to determine the specific differences. Differences between pre-MVC and post-MVC responses to ES during different time segments (pre, during TT and resting condition) for the same variable were analysed using the General Linear Model. However, differences between pre-MVC and post-MVC values at one occasion were analysed using one way ANOVA. Correlation coefficients between PT and RTD, and between PT and RR were performed, and r^2^ values were presented. The statistical significance was defined at p <0.05.

## Results

Total exercise duration was 347 ± 98 s for the 30,000 J. Average peak torque per extension–flexion cycle during the TT was 54 ± 13% of the maximal concentric torque measured pre-TT. Isometric MVC torque decreased significantly (p <0.001) by −48 ± 11% of the baseline value at the end of the TT and recovered significantly (p <0.05) after 2 min of rest. Those results and peak evoked torque responses without comparisons to “unpotentiated” measurements have been described in the study by Froyd et al. (2013b).

### Changes in absolute and relative peak evoked torque

**Figure 2:**
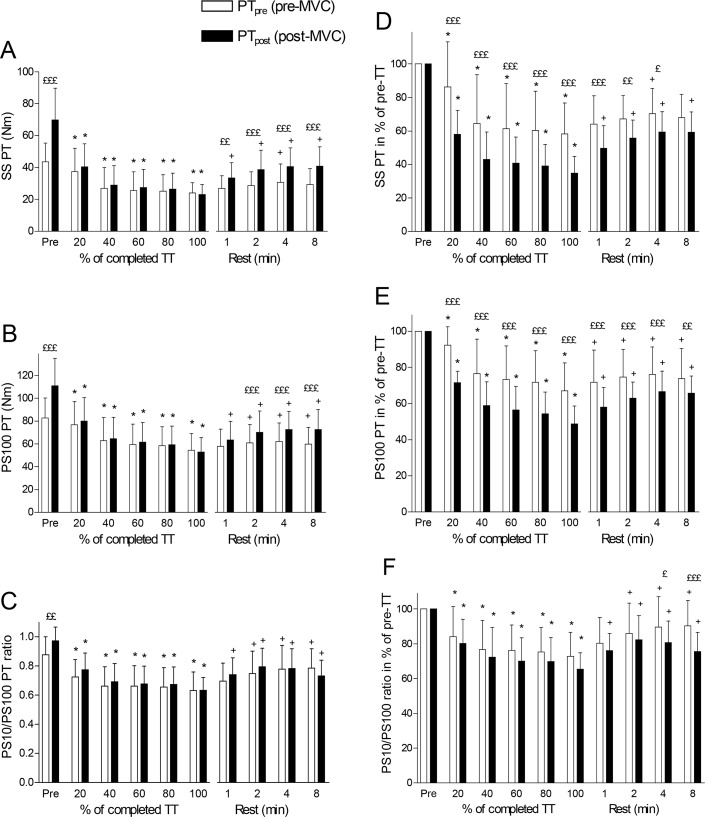
Absolute and relative peak evoked torque (PT) response to electrical stimulation for pre-MVC (PT_pre_) and post-MVC (PT_post_) single stimulus (SS) (A and D), paired stimuli at 100 Hz (PS100) (B and E), and PT ratio for paired stimuli at 10 Hz (PS10)/PS100 (C and F) before, during and for 8 min after a knee extension-flexion TT in a dynamometer. Values are expressed as means ± SD, n = 10. Significant differences between PT_pre_ and PT_post_: ££ p <0.01; £££ p <0.001; significant difference from pre-values for the same variable during the TT: * p <0.05; significant difference from 100% (end of TT) for the same variable during rest: ^+^ p <0.05.

Prior to exercise (TT), PT increased significantly (p <0.001) by 60 ± 15%, 34 ± 5% and 51 ± 21%, respectively, when measured after MVC with SS (Figure 2A), PS100 (Figure 2B), or PS10 (data not shown), while the PS10/PS100 ratio increased significantly (p <0.01) by 12 ± 13% (Figure 2C). PS100 increased significantly less than SS (p <0.001) and PS10 (p <0.05). When measured with SS or PS100, both PT_pre_ and PT_post_ decreased after 20% of the TT. There were no differences between PT_pre_ and PT_post_ at any time during the TT for SS or PS100 (Figure 2A and 2B). After 1 min of recovery, the PT_post_ response to both SS and PS100 increased significantly. The recovery of PT_pre_ was slower and increased significantly only 2–4 min after exercise termination in response to SS or PS100. Figure 2C shows that both PS10/PS100_pre_ and PS10/PS100_post_ ratios fell equally during exercise and increased similarly during the rest period, but did not reach pre-exercise levels 8 min after termination of exercise.

The extent to which SS or PS100 identified changes in PT_pre_ and PT_post_ as percentage of the corresponding pre-TT values is shown in Figure 2D–F. Correcting the pre-TT values to 100% adjusts for the pre-TT effect of potentiation. Figure 2D shows a significant difference in the extent to which PT_pre_ and PT_post_ falls (reduced by −42 ± 18% and −65 ± 10%, respectively at termination of the TT) for SS; Figure 2E shows the same information in response to PS100 (reduced by −33 ± 15% and −51 ± 10%, respectively at termination of the TT). In both cases PT_post_ was significantly (p <0.001) more reduced than PT_pre_ (SS: −40 ± 7%, PS100: −27 ± 7%, p <0.001). The PS10/PS100 ratio shows the same general pattern as for SS and PS100. However, the responses of PS10/PS100_pre_ and PS10/PS100_post_ ratios were not significantly different.

### PT_post_ and PT_pre_ between SS and PS100 during and after exercise

**Figure 3:**
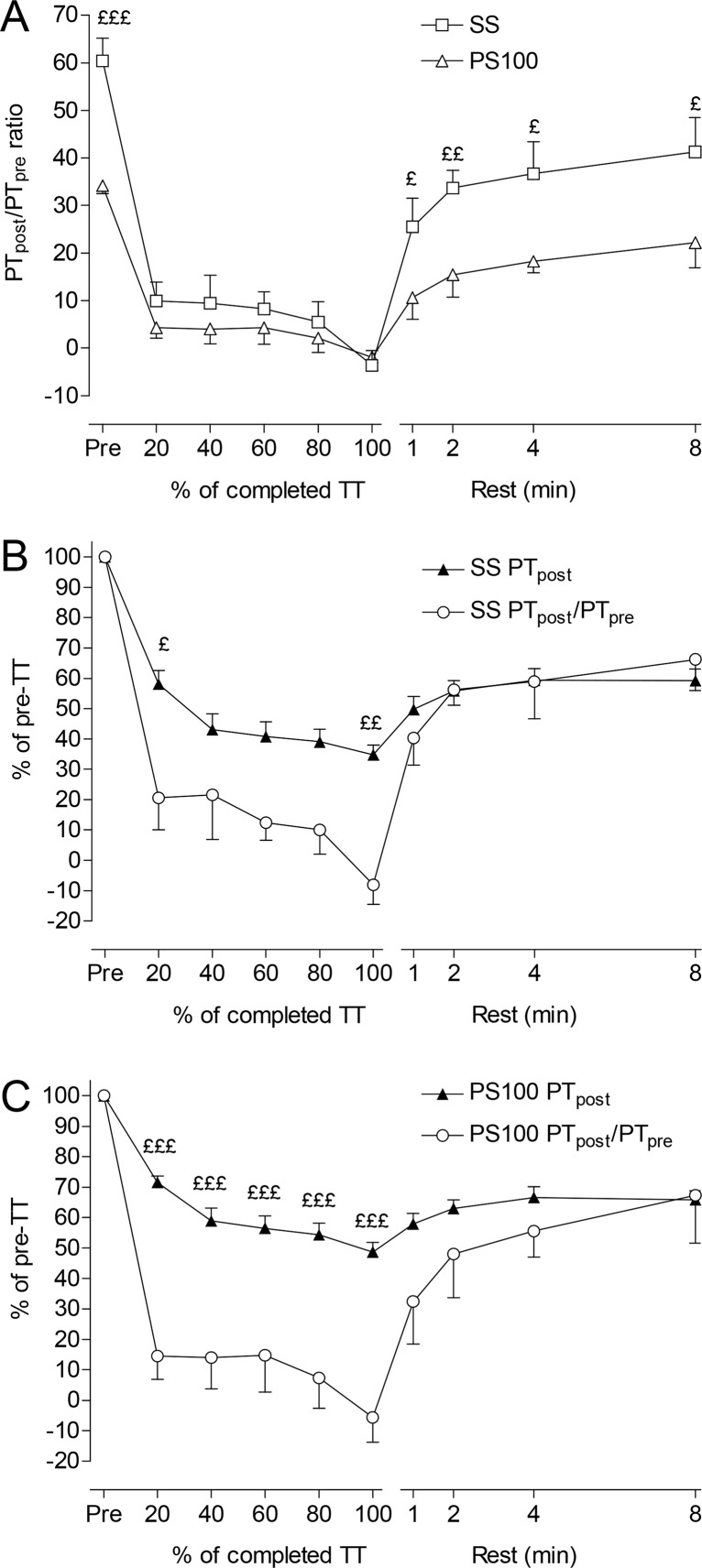
Peak evoked torque post-MVC (PT_post_) divided by pre-MVC (PT_pre_) for single stimulus (SS) and for paired stimuli at 100 Hz (PS100) shown as PT_post_/PT_pre_ ratio (A), PT_post_/PT_pre_ ratio and PT_post_ as percentage of pre-TT for SS (B), and PT_post_/PT_pre_ ratio and PT_post_ as percentage of pre-TT for PS100 (C), before, during and for 8 min after a knee extension-flexion TT in a dynamometer. Values are expressed as means ± SEM, n = 10, significant differences between SS and PS100 in panel A, and between PT_post_/PT_pre_ ratio and PT_post_ in panel B and C: £ p <0.05; ££ p <0.01; £££ p <0.001.

The PT_post_/PT_pre_ ratio was significantly (p <0.05) less for PS100 compared to SS prior to and after the TT (Figure 3A). However, during exercise none of the stimulation methods detected any effect of MVC on PT. From similar PT_post_ and PT_pre_ values at TT termination for both SS and PS100, after 1 min recovery the PT_post_/PT_pre_ ratio was significantly greater (p <0.05) for SS (25 ± 19%) compared to PS100 (11 ± 14%) (Figure 3A). Figures 3B and 3C show the changes in PT_post_ as an indicator of fatigue, and PT_post_/PT_pre_ as an index of potentiation for SS and PS100, respectively. Changes in PT correlated with changes in PT_post_/PT_pre_ for SS (r^2^ = 0.81, p <0.001) and PS100 (r^2^ = 0.69, p <0.01).

### Relationship between changes in PT, RTD and RR during and after exercise

**Figure 4:**
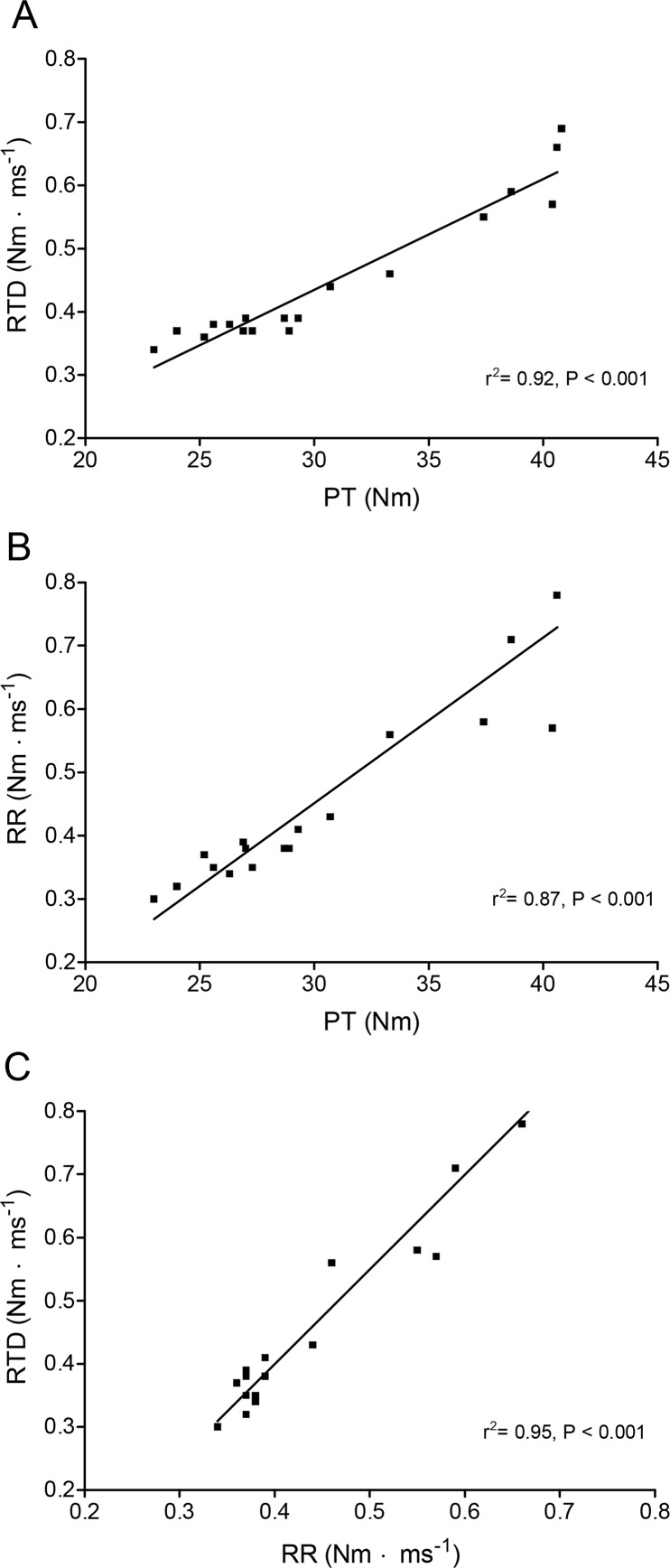
Correlation between peak evoked torque (PT) and rate of torque development (RTD) (A), PT and rate of relaxation (RR) (B), and RTD and RR (C) for all pre-MVC and post-MVC measurements during and after the time trial. Values are expressed as means for all subjects, n = 10.

RTD and RR increased significantly (p <0.001) after a MVC before the TT (data not shown). Thereafter RTD_pre_ and RTD_post_ in addition to RR_post_ and RR_pre_ changed in a relatively similar way as PT (Figure 2A) during exercise and recovery. The relationship between PT and RTD, PT and RR, and RTD and RR during and after exercise for both pre-MVC and post-MVC values (r^2^ = 0.92, p <0.001, r^2^ = 0.87, p <0.001, and r^2^ = 0.95, p <0.001, respectively) are shown in Figures 4A, 4B and 4C. The strong correlation between these variable existed not only for the entire experiment, but also separately during or after the exercise or for pre-MVC or post MVC measurements separately (data not shown).

## Discussion

The first finding of the present study was that a MVC potentiated the response to SS more than to PS100 prior to and as early as 1 min after the TT (Figure 3A). As far as we know, this is the first study to make this comparison during and after intense dynamic exercise. We concluded that SS could both be more potentiated and affected by fatigue than PS100 (Figures 2A–C and 3A). Since potentiation is an issue that must be controlled during fatigue measurements, PS100 may therefore be beneficial compared to SS for measurements of fatigue in exercise. The difference between SS and PS100 can be related to the shape of the force/frequency relationship (Edwards et al., 1977). Reduced PT during exercise may be related to decreased intracellular [Ca^2+^] and reduced myofibrillar Ca^2+^-sensitivity (Allen et al., 2008).

In Froyd et al. (2013b) it is shown that the extent to which PT falls during exercise is a function of the stimulation method, is greater with SS than with PS100 and is least with tetanic stimulation. Those results were collected immediately after a MVC to maximize potentiation of PT. In the present paper, we show that these differences between stimulation methods also exist when comparing PT before and after a MVC. The potentiation by a MVC disappeared as fatigue occurred and the time course of fatigue and potentiation was correlated. The correlation between potentiation and fatigue suggests that these two opposing phenomena are related. It is suggested that inorganic phosphate (Pi), which increases in the muscle during exercise, may both decrease intracellular [Ca^2+^] and myofibrillar Ca^2+^-sensitivity, which has been demonstrated in single animal muscle fibres (Millar and Homsher, 1990; Allen et al., 2008; Allen, 2013).

It is speculated that the same biological mechanisms that cause potentiation (changes in Ca^2+^ sensitivity) may also explain the development and recovery of peripheral fatigue since intracellular [Ca^2+^] is first decreased during exercise and then reversed during recovery. Several factors may influence Ca^2+^ sensitivity and intracellular [Ca^2+^], but RLC phosphorylation is thought to be the most important in affecting Ca^2+^ sensitivity (Macintosh, 2003). Although intracellular acidosis is often related to fatigue, it does not seem to reduce force production during exercise (Allen et al., 2008).

If correct, this interpretation supports the existence of a “peripheral governor” in skeletal muscle (Macintosh and Shahi, 2011; Westerblad et al., 2010) and is compatible with a key role for Ca^2+^ handling (Jones et al., 2009) and perhaps RLC phosphorylation (Sweeney et al., 1993; Macintosh et al., 2012) in these processes. This “peripheral governor” would act as a regulatory process to avoid ATP disturbance or metabolic catastrophe by decreasing Ca^2+^ release and thereby attenuating use of ATP by both Ca^2+^ ATPase and myosin ATPase (Macintosh and Shahi, 2011).

The next finding was that changes in PT were significantly correlated with RTD and RR during and after exercise (Figures 4A–B) as also found by others during exercise (Dolmage and Cafarelli, 1991). We (Froyd et al., 2013a) and others (Paasuke et al., 2007; Requena et al., 2008) have shown that the increase in PT caused by potentiation is associated with increases in both RTD and RR. In addition, a significant correlation between RTD and RR (Figure 4C) indicates that mechanisms related to torque development are also related to torque relaxation. This suggests that processes related to Ca^2+^-release from sarcoplasmic reticulum change similarly with Ca^2+^ pumping back to sarcoplasmic reticulum.

The last finding was that the calculation quantifying the extent of the fall in PT during exercise was influenced by the presence or absence of a MVC to potentiate PT before exercise. Figures 2D–E show that whereas PT_pre_ fell to approximately 55% and 70% of pre-TT values at the termination of the TT, PT_post_ decreased much more since PT_post_ during exercise was probably already potentiated by the TT exercise. A MVC during a TT may cause both potentiation and fatigue, but we interpreted the results (Figures 2A–B) assuming that RLC phosphorylation was already high prior to the MVC during the TT. However, the potentiating effect of the MVC was re-established already within 1–2 min after the termination of exercise (Figures 2A–B). The difference seen between PT_post_ and PT_pre_ before exercise confirms previous findings that a potentiated twitch is a better and more sensitive measure of peripheral fatigue than unpotentiated twitch (Alway et al., 1987; Kufel et al., 2002; Place et al., 2007). However, the originality of the present study compared to other studies is that PT_post_ and PT_pre_ are measured not only prior to or after exercise, but also during and in the recovery phase after exercise. Always et al. (1987) found no reductions in PT_post_ or PT_pre_ after exercise despite a 35% reduction in a MVC, and hence PT_post_/PT_pre_ was unchanged after exercise. This contrasts with the present study showing decreased PT_post_/PT_pre_ during exercise and recovery of PT_post_/PT_pre_ after exercise (Figure 3A).

The practical relevance of these findings is that studies that do not measure pre-exercise PT in potentiated muscles or that delay the post-exercise measurements of PT by more than a few seconds, will underestimate the extent to which peripheral fatigue develops. Such studies may conclude that central fatigue plays a larger role in impairing performance during exercise (Millet et al., 2002; Saldanha et al., 2008) than will studies that include these experimental methods (Matkowski et al., 2011). Although we did not measure central fatigue in this study, the levels of peripheral fatigue that we have documented are amongst the highest reported in the literature. This may suggest that the extent to which peripheral fatigue develops during exercise has been generally underestimated by previous studies.

The consequence for studies of NMF is that measures of PT that are not potentiated by a MVC will not detect the true nature of the recovery process that occurs in potentiated muscles after exercise. This confirms the conclusion from Kufel et al. (2002) and may explain the slow recovery for PT in other studies which used “unpotentiated” post exercise measurements (Behm and St-Pierre, 1997; Place et al., 2004; Lattier et al., 2004). However, in contrast to Kufel et al. (2002) who measured the effect of potentiation 15 minute after exercise, we compared the effect of potentiation immediately after the termination of exercise.

In conclusion, this study shows that potentiation influences the peak evoked torque response differently depending on the applied electrical stimulation method. Peak evoked torque was more potentiated by a MVC for SS than PS100 before and after, but not during exercise since no extra potentiation was observed during the intense dynamic exercise. More fatigue was measured during and after exercise when ES was applied after compared to before a 5 s MVC. In addition, the present findings suggest that potentiated measurements recovered faster than unpotentiated measurements after exercise and that SS can be more potentiated and fatigued than PS100. Studies that do not measure pre- or post-exercise peak evoked torque in muscles potentiated by a MVC or that delay the post-exercise measurements of peak evoked torque more than a few seconds, will underestimate the extent to which peripheral fatigue develops, and the underestimation will be more pronounced for SS compared to PS100. This may explain the slow recovery for peak evoked torque in other studies which used “unpotentiated” single stimulus after exercise.
